# Support Vector Machine Based on Adaptive Acceleration Particle Swarm Optimization

**DOI:** 10.1155/2014/835607

**Published:** 2014-03-25

**Authors:** Mohammed Hasan Abdulameer, Siti Norul Huda Sheikh Abdullah, Zulaiha Ali Othman

**Affiliations:** ^1^Pattern Recognition Research Group, Centre for Artificial Intelligence Technology, Faculty of Information Science and Technology, Universiti Kebangsaan Malaysia, 43600 Bandar Baru Bangi, Malaysia; ^2^Department of Computer Science, Faculty of Education for Women, University of Kufa, Iraq; ^3^Data Mining and Optimization Group, Centre for Artificial Intelligence Technology, Faculty of Information Science and Technology, Universiti Kebangsaan Malaysia, 43600 Bandar Baru Bangi, Malaysia

## Abstract

Existing face recognition methods utilize particle swarm optimizer (PSO) and opposition based particle swarm optimizer (OPSO) to optimize the parameters of SVM. However, the utilization of random values in the velocity calculation decreases the performance of these techniques; that is, during the velocity computation, we normally use random values for the acceleration coefficients and this creates randomness in the solution. To address this problem, an adaptive acceleration particle swarm optimization (AAPSO) technique is proposed. To evaluate our proposed method, we employ both face and iris recognition based on AAPSO with SVM (AAPSO-SVM). In the face and iris recognition systems, performance is evaluated using two human face databases, YALE and CASIA, and the UBiris dataset. In this method, we initially perform feature extraction and then recognition on the extracted features. In the recognition process, the extracted features are used for SVM training and testing. During the training and testing, the SVM parameters are optimized with the AAPSO technique, and in AAPSO, the acceleration coefficients are computed using the particle fitness values. The parameters in SVM, which are optimized by AAPSO, perform efficiently for both face and iris recognition. A comparative analysis between our proposed AAPSO-SVM and the PSO-SVM technique is presented.

## 1. Introduction

Support vector machine (SVM) is a machine-learning method based on the structure risk minimization principle. SVM can find global optimum solutions for problems with small training samples, high dimensions, and nonlinearity. SVM has attracted much attention during the past decade as a modern machine-learning approach in several domains, such as pattern recognition, bioinformatics, and other nonlinear problems with small sample sizes. SVM has strong theoretical foundations and a good generalization capability. From the implementation point of view, training an SVM in classification is equivalent to solving a linearly constrained quadratic programming (QP) problem, which consumes large amounts of memory and computation time when the number of samples increases. Another issue in SVM is that the selection of the training parameters impacts its performance. Some of the SVM-based methods are utilized with face applications [1–15]. Li, Lijuan & Weiguo have proposed a multi-class SVM for face recognition [2]. They utilized the generalized two-dimensional Fisher's linear discriminant (G-2DFLD) method for feature extraction and used multiclass support vector machines as the classifier for face recognition. They proposed a multiobjective uniform design (MOUD) search method as an SVM model selection tool and then applied an optimized SVM classifier to face recognition. In their proposed method, LDA has been used for feature extraction. However, LDA is computationally high and suffers from the so-called small size problem (SSS) problem [13]. Additionally, a new classification model based on SVM named as (SVM + NDA) has been proposed by Khan et al. [12]. In addition, they proposed a kernel extension of the model KSVM + KNDA to deal with nonlinear problems. However, it is obvious to notice that the SVM + NDA in linear case took more computational time than the LDA, NDA, HLDA, and SVM + LDA. On the other hand, the KSVM + KNDA required greater number of iterations than KNDA and KFD in nonlinear case.

Recently, existing face recognition methods utilize PSO and OPSO methods to optimize the parameters of SVM. Reference [3] presented a face recognition method based on support vector machine and particle swarm optimization (PSO-SVM). In PSO-SVM method, the parameters optimization problem in SVM is solved by particle swarm optimization. Nevertheless, this method lacks the initial phase of the PSO technique. In PSO, the populations are generated in a random manner. Due to this random process, the population results may also be in a random manner. Therefore, it is not certain that this method will produce a precise result when it is used with SVM. Later, and to avoid this drawback, a modified face recognition method based on opposition particle swarm optimization (OPSO) and SVM (OPSO-SVM) has been proposed by Hasan, Abdullah and Othman [13]. In OPSO-SVM, opposition particle swarm optimization (OPSO) [14] has been used instead of PSO to find the optimal parameters in SVM. In OPSO, the populations are generated in two ways: one is random population the same as the standard PSO technique and the other is opposition population, which is based on the random population values. The optimized parameters in SVM by OPSO efficiently perform the face recognition process. Accelerated PSO with SVM (APSO-SVM) has been introduced by Yang, Deb and Fong [15]. In APSO-SVM, APSO is used to find the best kernel parameters in SVM. Then, the kernel parameters are used to construct the support vector machines to solve the problem of interest. In APSO, the algorithm used the global best only and excluded the individual best and it does not use velocities or inertia parameter. Though, the PSO performance degrades by the utilization of random values in the velocity calculation and this will influence the parameter selection in SVM. In this paper, to address this problem, an adaptive acceleration particle swarm optimization (AAPSO) technique is proposed.

The rest of this paper is structured as follows.  Section 2 describes the proposed model in relation to PSO and SVM.  Section 3 explains the experimental results. Finally, we end our paper with conclusions and potential future studies in Section 4.

## 2. The Proposed Model

**Figure 1:**
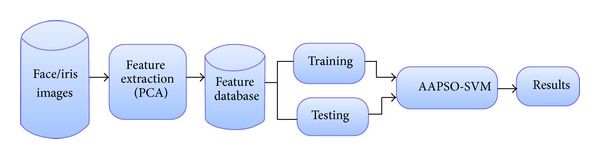
Structure of the proposed recognition technique based on AAPSO-SVM.

The standard PSO method has been utilized in many research works to obtain optimal problem solutions. To obtain a more accurate optimal result, the drawbacks which are present in the PSO method must be addressed by making modifications or enhancements to the PSO model. The major drawback of the PSO is the random value selection during new particle generation; that is, in the velocity computation, the acceleration coefficients are generated randomly. The random value selection in the velocity process means that the generated particles will also be random. Random populations do not produce more accurate results. Hence, to acquire a more accurate result and to reduce this PSO drawback, we propose an adaptive acceleration particle swarm optimization (AAPSO). To obtain more accurate classification results, the SVM parameters will be optimized by our AAPSO. The utilization of AAPSO in the SVM parameter optimization will reduce the PSO drawback and improve the classification result accuracy. In this research, our intent is to develop a face and iris recognition system for accurate recognition of face images from the databases. The proposed face recognition technique is performed in three phases, feature extraction by PCA, adaptive acceleration particle swarm optimization (AAPSO), and parameters selection for SVM with AAPSO. These three phases are performed repeatedly on the input database face images, and thus the face images are recognized more effectively. The three phases are discussed in Sections 2.1, 2.2, and 2.3. The basic structure of our proposed face recognition technique is shown in Figure 1.

### 2.1. Feature Extraction Using PCA

The purpose of the feature extraction is to extract the information that represents the face. Principal component analysis (PCA) is used for this purpose [9]. We apply PCA on the training and testing database face images and obtain the unique dimensional feature vectors.

### 2.2. The Proposed Adaptive Acceleration Particle Swarm Optimization (AAPSO)

Particle swarm optimization (PSO) is a computational intelligence oriented, stochastic, population based global optimization technique proposed by Kennedy and Eberhart [4, 11]. It is inspired by the social behaviour of biological creatures, such as fishes and birds, which have the ability to group together to work as a whole to locate desirable positions in a certain area, for example, fish searching for a food source. This type of search behaviour is equivalent to searching for solutions of equations in a real-valued search space [10]. PSO emulates the swarm behaviour of individuals who represent potential solutions in a D-dimensional search space. Particle *i* is often composed of four vectors: *X*_*i*_ = (*x*_*i*_^1^, *x*_*i*_^2^,…, *x*_*i*_^*D*^), where *x*_*i*_^*D*^ is its position in the *d*th dimension; *Pbest*_*i*_ = (*Pbest*_*i*_^1^, *Pbest*_*i*_^2^,…, *Pbest*_*i*_^*D*^), where *Pbest*_*i*_^*D*^ is the best position in the *d*th dimension that particle *i* has found on its own; *V*_*i*_ = (*v*_*i*_^1^, *v*_*i*_^2^,…, *v*_*i*_^*D*^), where *v*_*i*_^*D*^ is the velocity in the *d*th dimension; and *gbest*_*i*_ = (*gbest*_*i*_^1^, *gbest*_*i*_^2^,…, *gbest*_*i*_^*D*^), where *gbest*_*i*_^*D*^ is the global best position in the *d*th dimension that all particles have found. Particles in a swarm move through the search space as follows:

(1) $$\begin{array}{c} {V_{i}}^{d}={V_{i}}^{d}+c_{1}r_{1}\cdot \left(Pbest_{i}^{d}-x_{i}^{d}\right)+c_{2}r_{2}\cdot \left(gbest_{i}^{d}-x_{i}^{d}\right), \end{array}$$

(2) $$\begin{array}{c} x_{i}^{d}=x_{i}^{d}+\delta V_{i}^{d}, \end{array}$$

where *c*_1_ and *c*_2_ are two constants, often with the value of 2.0, *r*_1_ and *r*_2_ are two independent random numbers uniformly generated in the range [0.1] at each updating iteration from *d* = 1 to *D*, *V*_*i*_^*d*^ is the velocity of the *i*th particle, *x*_*i*_^*d*^ is the current position of the particle *i*, *Pbest*_*i*_^*d*^ is the position of the best fitness value of the particle at the current iteration, and *gbest*_*i*_^*d*^ is the position of the particle with the best fitness value in the swarm. The random values of *c*_1_ and *c*_2_ in the velocity computation do not select the optimal SVM parameters so that the result of the recognition results will be random or inaccurate. Therefore, we have proposed an adaptive acceleration particle swarm optimization (AAPSO) method that selects the acceleration coefficients using particle fitness values. The AASPO method selects the optimal SVM parameters and formulates the SVM to provide a more accurate face recognition result. The AAPSO's acceleration coefficients are determined as follows:

(3) $$\begin{array}{c} {nc}_{1}=\frac{2}{3}\left(c_{1\max}-c_{1\min}\right)\left(\frac{f_{\min}}{f_{\text{avg}}}+\frac{f_{\min}}{2_{f_{\max}}}\right)+c_{1\min},\\ {nc}_{2}=\frac{2}{3}\left(c_{2\max}-c_{2\min}\right)\left(\frac{f_{\min}}{f_{\text{avg}}}+\frac{f_{\min}}{2_{f_{\max}}}\right)+c_{2\min}, \end{array}$$

where *c*_1max⁡_ and *c*_1min⁡_ represent the minimum and maximum values of *c*_1_; *f*_min⁡_, *f*_avg_, and *f*_max⁡_ are the particle minimum, average, and maximum fitness values of the entire population; and *c*_2max⁡_ and *c*_2min⁡_ represent the minimum and maximum values of *c*_2_. By applying *nc*_1_ and *nc*_2_ in the velocity equation (1), the equation is updated as follows:

(4) Vid=Vid+nc1r1·(Pbestid−xid)+nc2r2·(gbestid−xid).

Utilizing the above equations, the velocity function acceleration coefficients are computed in AAPSO. The evaluation of the coefficients using the AAPSO equations enables the SVM to provide more accurate results.

**Example 1**

Assume that the population pool has five particles/individuals, *P*_*n*_ (for simplicity), and their fitness values are as follows: *P*_1_ = 0.5, *P*_2_ = 0.4, *P*_3_ = 0.2, *P*_4_ = 0.7, *P*_5_ = 0.6, and *f*_min⁡_ = *P*_3_.

**Figure 2:**
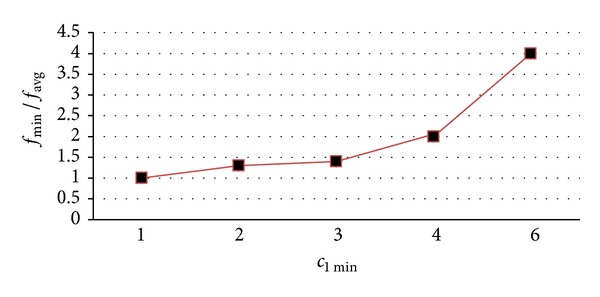
*c*_1 min⁡_ behavior.

As a special case, *f*_avg_ is equal to *f*_min⁡_ (i.e., *f*_min⁡_ = *f*_avg_) only if each member of the population has the same fitness value (i.e., *P*_1_ = *P*_2_ = *P*_3_ = *P*_4_ = *P*_5_). Otherwise, *f*_min⁡_ < *f*_avg_. Naturally, the probability of *f*_min⁡_/*f*_avg_ will be between 0 and 1. Assume that the probability of *f*_min⁡_/*f*_avg_ = 1 and the probability of *f*_min⁡_/*f*_max⁡_ = 1; then the sum ((*f*_min⁡_/*f*_avg_) + (*f*_min⁡_/2_*f*_max⁡__)) is 3/2. Therefore, we only restrict the value within 1, and (3) and (4) are multiplied by 2/3. As a result, this calculation will produce a maximum value of 1 instead of 3/2. If *f*_min⁡_, *f*_avg_, and *f*_max⁡_ are equal, the acceleration will be constant at 1; that is, *c*_1_ approaches *c*_1max⁡_. If *c*_1_ increases, then the neighbourhood search space diverges using PSO. Otherwise, the neighbourhood search space converges. That is, *c*_1_ is linearly proportional to its neighbourhood search space. The data in Figure 2 show that *c*_1 min⁡_ varies linearly with*f*_min⁡_/*f*_avg_.

The acceleration constant, *c*_1_, accelerates the neighborhood search; that is, it determines the nearest or farthest one. If *c*_1_ is small, the updated solutions will be near to the current solution, whereas if *c*_1_ is high, the updated solution will be far from the current solution. In our adaptive method, instead of fixing a constant *c*_1_, we increase or decrease *c*_1_ at every iteration. Therefore, the updated solutions may be far, near, or near-far.

**Example 2**

Assume a range of 10 and initial acceleration of 0; that is, the acceleration constant can vary within [0,9]. For example, if a vehicle starts at 0 km/h and its maximum speed is 100 km/h, according to the traffic, it will accelerate between 0 and 100 km/h. Thus, the range of the speed is 100 − 0 = 100 (*c*_1max⁡_ − *c*_1 min⁡_). In (3), we add *c*_1 min⁡_ to calculate the speed from the initial condition. The conventional method fixes the acceleration constant at one value; thus, the velocity of the particles is updated without considering the relation to population fitness. By making this constant adaptive, we increase or decrease the velocity based on the population fitness.

### 2.3. Parameter Selection for SVM with AAPSO

**Figure 3:**
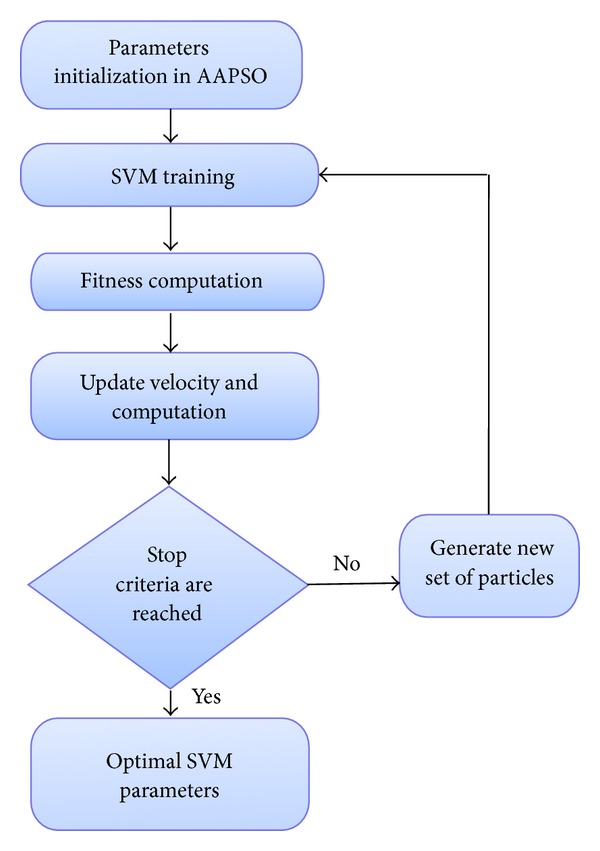
SVM parameter optimization using AAPSO.

To obtain more precise recognition, the SVM parameters are optimized using the AASPO method. The process is shown in Figure 3. The process of optimal parameter selection by AAPSO in SVM is shown as follows.

**Step 1**

Initially, the particles are generated randomly within the interval  [*x*, *y*]. The generated particles are composed of SVM parameters *P*_*i*_. Then, the parameters of each particle are initiated, including position and velocity.

**Step 2**

The fitness value of every particle is calculated using (5). The particles that have the minimum fitness values are selected as the best particles as follows:

(5)  min⁡ 12||Pi||2+C∑i=1Nξi

(6)  Such  that ∑i=1NPixi≥(1−ξiyi)−b,i=1,2,…,N,  ξi≥0,  i=1,2,…,N,

where  *N* is the size of the training dataset and *C* is a positive regularization constant or cost function, which defines the tradeoff between a large margin and a misclassification error.

**Step 3**

The $\overset{-}{{Pbest}_{i}}$ of each particle is updated and $\overset{-}{{gbest}_{i}}$ for the domain is updated. Based on these values, the velocity and position of every particle are updated using (4) and (2).

**Step 4**

Stop if the current optimization solution is good enough or if the stopping criterion is satisfied.

## 3. Experimental Results

**Figure 4:**
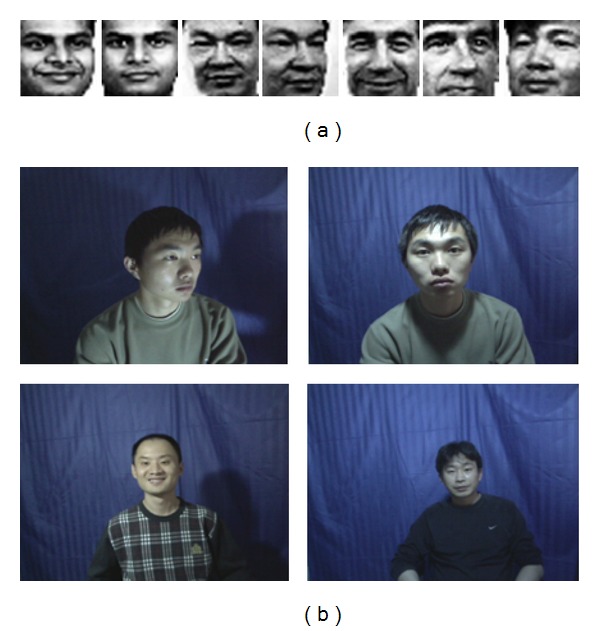
Sample face images from (a) YALE [5] and (b) CASIA [6] databases.

We divide our experiment into two sections, face recognition evaluation and then iris recognition evaluation. The proposed face recognition technique is implemented using MATLAB (version 7.12) on an Intel core i5 processor that uses Windows 7 operating system and that has 3.20 GHz CPU speed and 4 GB of RAM. The performance of the proposed face recognition technique is evaluated using the face databases YALE [5] and CASIA [6]. The images are obtained from both databases and the feature extraction is computed using PCA, while recognition process is computed using the proposed AAPSO-SVM technique. Sample face images from the YALE and CASIA databases are shown in Figure 4.

The performance of our proposed method is analyzed in three evaluation steps: (i) evaluate the optimization using three standard functions, (ii) evaluate the classification results with the UBiris dataset [7], and (iii) evaluate the classification results with the face datasets. These three evaluation steps are explained below.

*(i) Evaluate the Optimization Using Three Standard Functions*. To accomplish the performance analysis, we performed 10 rounds of experiments using the AAPSO and PSO methods. Moreover, our proposed AAPSO method performance is evaluated using the standard functions [8]: sphere, Rosenbrock, and Rastrigin. These standard functions are computed using the following equations:

(7) f0(x)=∑i=1nxi2,f1(x)=∑i=1n(100(xi+1−xi2)2+(xi−1)2),f2(x)=∑i=1n(xi2−10cos⁡(2Πxi)+10).

**Figure 5:**
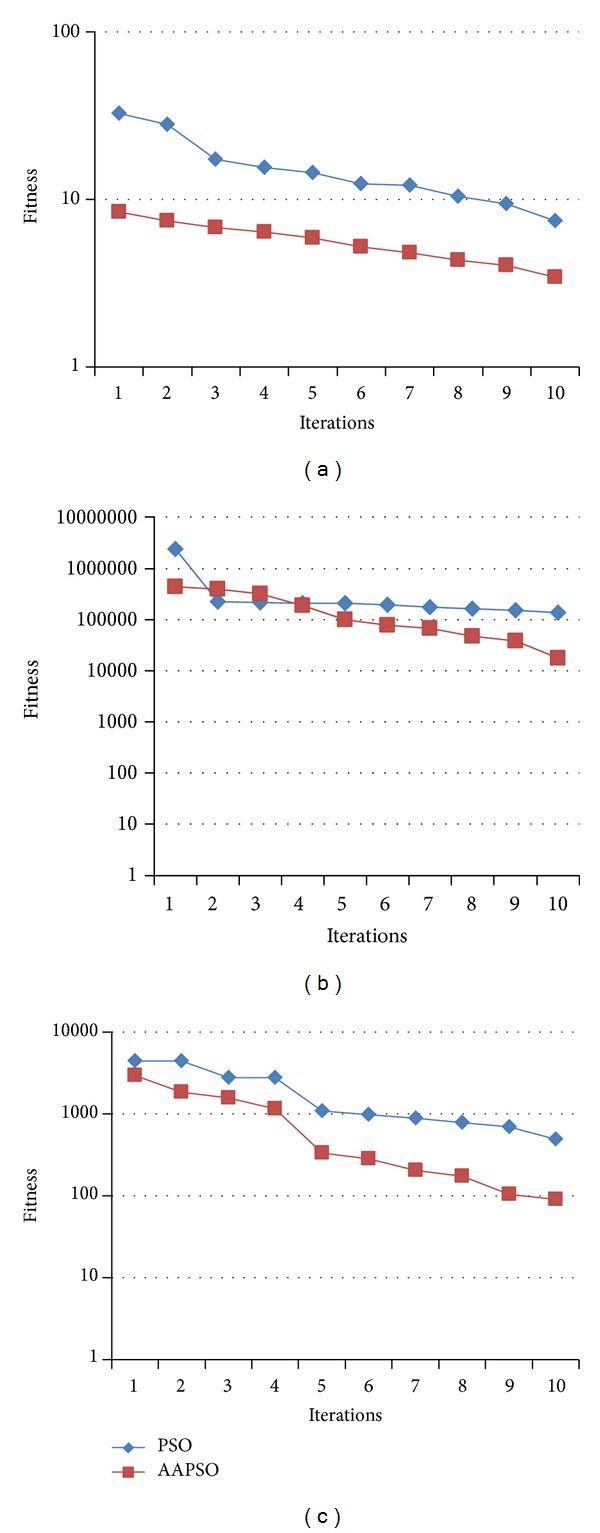
Performance of AAPSO and PSO methods with (a) sphere, (b) Rosenbrock, and (c) Rastrigin.

The performance of our proposed AAPSO and the standard PSO methods on these standard functions, in terms of their fitness values for different numbers of iterations, is shown in Figure 5.

**Figure 6:**
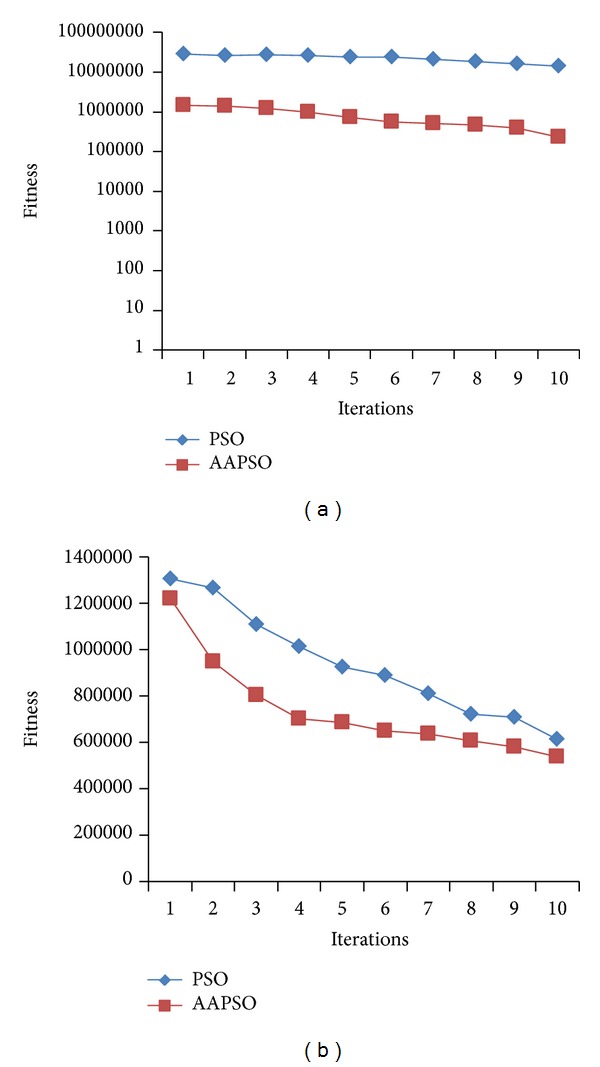
Performance of AAPSO and PSO methods from (a) YALE [5] database and (b) CASIA [6] database.

The results in Figure 5 show that our proposed AAPSO method yields more accurate particles that have lower fitness values than those generated by the PSO method. The results in Figures 5(a), 5(b), and 5(c) show that our proposed AAPSO method has obtained accurate fitness values for all of the three standard functions. The high performance result shows that our AAPSO method is able to determine the more accurate SVM parameters. Additionally, the performance of our proposed AAPSO method is compared to the performance of the PSO method using (5) in Figure 6.

In Figure 6, the proposed AAPSO technique obtained more accurate particles that have minimum fitness values smaller than those obtained with the PSO method. Therefore, our AAPSO technique has yielded more accurate SVM parameters.  Figure 6(a) shows the fitness value performance for particles used on the YALE database face images. For all iterations, the fitness values of the particles of our proposed AAPSO method are lower than those of the PSO method. However, when applied to the CASIA database, the PSO method particles have lower fitness values than the AAPSO particles for iterations 5, 8, and 9. In the remaining iterations, the AAPSO particles have the same or lower fitness values in comparison to the PSO particles.

**Figure 7:**
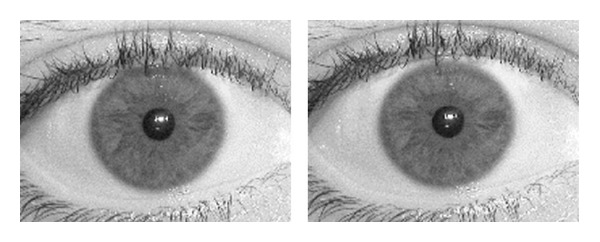
Sample images from the iris dataset.

*(ii) Evaluate Classification Results with the UBiris Dataset*. In this section, the classification performance is evaluated with the UBiris dataset. The classification accuracy results that are obtained for the UBiris dataset are given in Table 1. To analyze the classification performance, 10 experiments are conducted on the iris dataset. Sample iris dataset images are shown in Figure 7.

In 10 experiments, our proposed AAPSO method attained higher iris image classification accuracy than the standard PSO-SVM. The average classification accuracy is 95%.

**Figure 8:**
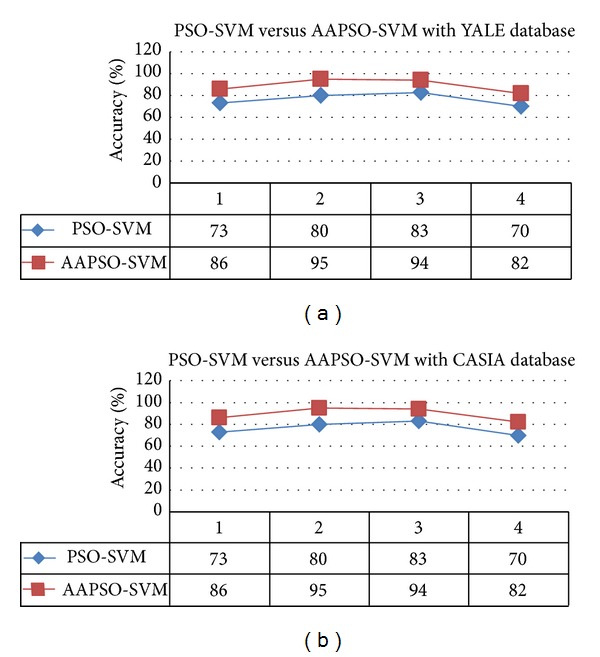
Performance accuracy of theAAPSO and PSO recognition methods from (a) YALE [5] database and (b) CASIA [6] database.

**Figure 9:**
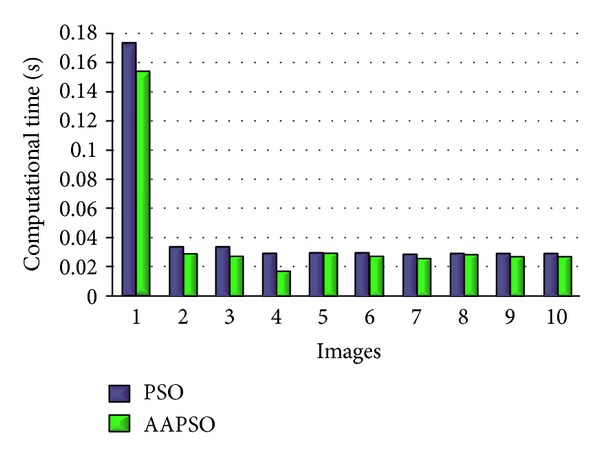
The computation time of AAPSO-SVM and PSO-SVM.

*(iii) Evaluate Classification Results with Face Datasets*. In this section, the classification results are evaluated with two databases, Yale and CASIA. Moreover, the performance of our proposed technique is compared with the PSO-SVM method using based on accuracy rate. In the experiment, the face images are evaluated for four conditions: (i) same pose, same illumination, and different expression; (ii) same pose, same expression, and different illumination; (iii) same expression, same illumination, and different pose; and (iv) different expression, pose, or illumination. The accuracy results for the proposed AAPSO-SVM and the existing PSO-SVM face recognition techniques, applied to the YALE and CASIA databases, with the different conditions, are shown in Table 2 and Figure 8. The computational times for our proposed AAPSO and for the PSO methods are shown in Figure 9.

As shown in Figure 8, the recognition results of AAPSO-SVM have higher face recognition accuracy than the results of the PSO-SVM in all of the four conditions for both the Yale and CASIA databases. We measured the *t*-test values for the accuracy between AAPSO-SVM and PSO-SVM face recognition techniques. There was not much variation in the *t*-test results; however, the *t*-test result shows that the proposed AAPSO-SVM is statistically significant and that it outperforms the PSO-SVM with the *t*-test result *P* < 0.05; *P* = 0.0265 for the Yale database and *P* = 0.0186 for the CASIA database. Furthermore, Figure 9 shows the computational time used by our proposed AAPSO method and the conventional PSO to determine the optimal SVM parameters. It was shown that AAPSO optimized the SVM when compared with conventional PSO.

Figure 9 shows the computational time used by our proposed AAPSO method and the conventional PSO to determine the optimal SVM parameters. It was shown that AAPSO optimized the SVM, when compared with conventional PSO. The results in the table demonstrate the computational efficiency of AAPSO. That is, AAPSO uses less computational time to perform the optimization process compared with the conventional PSO. On average, AAPSO used 12% of the computational time to optimize the parameters and PSO used 21% of the computational time to optimize the parameters.

## 4. Conclusions 

In this paper, we introduced AAPSO based on SVM to address the limitation of the standard PSO method that uses random selection of the coefficient factor for velocity. This may lead to performance instability. The optimized SVM, using the AAPSO technique, shows effective face recognition performance. Two human face databases, YALE and CASIA, were utilized to analyze the performance of our proposed AAPSO-SVM face recognition technique. The UBiris database was also used to illustrate the performance of our proposed technique in other domains. The performance and comparative analysis results show that our proposed AAPSO-SVM technique yields higher face recognition performance results than the PSO-SVM face recognition methods. In 10 experiments, our proposed AAPSO method attained a high iris image average classification accuracy of 95%, which is more than the standard PSO-SVM, which attained 90% in the same experiments. In addition, the SVM parameters for the YALE and CASIA databases are more optimal when obtained from AAPSO than from the conventional PSO. AAPSO also takes less computational time to perform the optimization process than the conventional PSO. On average, AAPSO used 12% of the computational time to optimize the parameters and PSO used 21% of the computational time to optimize the parameters. Hence, our proposed AAPSO with SVM technique is more robust and more precisely recognizes the face and iris images. Our proposed method can be made more robust if we test it with domains other than biometrics, such as bioinformatics and text categorization.

## Figures and Tables

**Table 1:** The accuracy of PSO and AAPSO based on SVM classification performance results for the UBiris dataset.

Experiment number	Accuracy (%) PSO	Accuracy (%) AAPSO
1	90	95
2	87	93
3	91	94
4	90	94
5	92	96
6	89	96
7	91	94
8	92	95
9	88	94
10	92	95

Average	90	95

**Table 2:** Accuracy values of the proposed AAPSO-SVM and the PSO-SVM techniques with face datasets.

Conditions	Condition description	Yale dataset		CASIA dataset	
		Method	Accuracy	Method	Accuracy
1	Same pose, same illumination, and different expression	PSO-SVM	73	PSO-SVM	76
		AAPSO-SVM	86	AAPSO-SVM	85
2	Same pose, same expression, and different illumination	PSO-SVM	80	PSO-SVM	82
		AAPSO-SVM	95	AAPSO-SVM	96
3	Same expression, same illumination, and different pose	PSO-SVM	83	PSO-SVM	81
		AAPSO-SVM	94	AAPSO-SVM	93
4	Different expression, pose, or illumination	PSO-SVM	70	PSO-SVM	75
		AAPSO-SVM	82	AAPSO-SVM	84
